# Jubilee Meeting of American Medical Association

**Published:** 1897-07

**Authors:** 


### EDITORIAL.

The office of the Business Manager of THE JOURNAL is 311 and 312 Fitten Building.

The editors are not responsible for views expressed by contributors.

Articles for publication should reach this office not later than the 15th inst.

Address all communications, and make all remittances payable to THE ATLANTA MEDICAL AND SURGICAL JOURNAL, Atlanta, Ga.

Reprints of articles will be furnished, when desired, at a small cost. Requests for the same should always be made *on the manuscript*.

### JUBILEE MEETING OF AMERICAN MEDICAL ASSOCIATION.

We reached Philadelphia on May 31st, so as to be in good time for the meeting of the American Medical Editors Association. This association does not receive the support to which it is entitled. Every editor of a reputable medical journal in the United States should be a member, and an effort will be made by the newly elected officers to bring them into the fold. The meetings are not only valuable, they are exceedingly enjoyable. It is very pleasant to meet socially, and hear talk, and look into the eyes of an editor whose journal you have read with interest for years.

The annual banquet, held at Hotel Aldine, on the evening of June 1st, was a delightful success, and was participated in by representatives from almost every section of the country. But I must not take up too much space with the editors when there is so much else of interest to write about the Jubilee Meeting of the American Medical Association.

The first day's general session was made notable by the able address of the president, Dr. Nicholas Senn. Full of interest and valuable suggestions from beginning to end, without a dull paragraph. It ought to be read by every physician in America.

The second day by the scholarly address of Dr. Austin Flint. The third day by the beautiful, the scholarly, the magnificent address on Surgery by Dr. W. W. Keen. No man who loves surgery could help receiving the liveliest pleasure from listening to his interesting array of the brilliant achievements of surgery, and his sanguine predictions of still greater achievements for the future. And also by the Jubilee exercises conducted by that grand old man, the father of the Association, Dr. N. S. Davis, of Chicago. The fourth by the scientific address by Dr. John B. Hamilton, on State Medicine.

The report of the Rush monument committee elicited a great deal of interest, and quite a large sum of money was raised with which to build a suitable monument. Several of the more populous States guaranteed to raise at least $2,000. A number of individual subscriptions of $100 were also made. There is now hope that a suitable monument will be erected to this greatest of America's physcian-statesmen.

The scheduled hospital work the week before, and the week after the meeting was more than worth the trip, and demonstrated the great interest which the profession of Philadelphia took in making the meeting a success.

The entertainments and receptions were many and varied, and to Dr. Hobart A. Hare, the chairman of the committee of arrangements, is due the unstinted praise and hearty thanks of the Association for the unprecedented variety and brilliance of the entertainments. But there is danger of too much attention being given to this feature of the meetings. Nothing should be allowed to interfere with the regular work of the general sessions and the sections.

Another danger is that too many papers are read before the sections. Too little discrimination is shown by the officers of the sections in arranging the program; so many papers are read that there is very little time left for discussing the most valuable part of the work. There ought to be a better grouping of the papers, and the president should appoint able men to open the discussions on papers and subjects of special interest.

Denver was selected as the next place of meeting, and the Association was put on notice by the chairman of the committee of arrangements that over $10,000 had already been procured by subscriptions with which to entertain the Association.

Never in the history of the Association has there been held a meeting fraught with so much interest to the American medical profession. Never have so many able and epoch-making addresses been delivered before any medical body in the New World. Never was the Association so united and earnest in its efforts to raise the professional standard, and to elevate the character of men who have the health and lives of their fellow-beings in their hands. Never was there a time when members of the regular profession could so ill afford not to be members of the great American Medical Association.

The following officers were elected for the current year:

President—Geo. M. Sternberg, Surgeon-General, U. S. A., Washington, D. C.

First Vice-President—Joseph M. Mathews, Louisville, Ky.

Second Vice-President—J. L. Thompson, Indianapolis, Ind.

Third Vice-President—F. H. Wiggin, New York, N. Y.

Fourth Vice-President—T. J. Happel, Trenton, Tenn.

Treasurer—Henry P. Newman, Chicago, Ill.

Secretary—Wm. B. Atkinson, Philadelphia, Pa.

Assistant Secretary—W. A. Jayne, Denver, Colo.

Chairman Committee of Arrangements—J. W. Graham, Denver, Colo.

The address on Medicine at the next meeting will be delivered by Dr. J. H. Musser, of Philadelphia; the address on Surgery by Dr. J. B. Murphy, of Chicago, and the address on State Medicine by Dr. S. C. Busey, Washington, D. C. F. W. M.

